# Impact of the COVID-19 pandemic on cardiac implantable electronic device implantation in China: Insights from 2 years of changing pandemic conditions

**DOI:** 10.3389/fpubh.2022.1031241

**Published:** 2022-11-22

**Authors:** Chen-di Cheng, Shuang Zhao, Jiang Jiang, Na Lin, Ping Li, Xiao-hui Ning, Shu Zhang

**Affiliations:** ^1^State Key Laboratory of Cardiovascular Disease, Arrhythmia Center, National Center for Cardiovascular Diseases, Fuwai Hospital, Chinese Academy of Medical Sciences and Peking Union Medical College, Beijing, China; ^2^National Center for Cardiovascular Quality Improvement, Fuwai Hospital, National Center for Cardiovascular Diseases, Beijing, China

**Keywords:** COVID-19, cardiac implanted electronic device (CIED), healthcare resource, secondary hospital, telemedicine, post COVID-19

## Abstract

**Background:**

A substantial reduction in the number of cardiac implantable electronic device (CIED) implantation was reported in the early stages of the COVID-19 pandemic. None of the studies have yet explored changes in CIED implantation during the following pandemic.

**Objective:**

To explore changes in CIED implantation during the COVID-19 pandemic from 2020 to 2021.

**Methods:**

From 2019 to 2021, 177,263 patients undergone CIED implantation from 1,227 hospitals in China were included in the analysis. Generalized linear models measured the differences in CIED implantation in different periods. The relationship between changes in CIED implantation and COVID-19 cases was assessed by simple linear regression models.

**Results:**

Compared with the pre-COVID-19 period, the monthly CIED implantation decreased by 17.67% (95% CI: 16.62–18.72%, *p* < 0.001) in 2020. In 2021, the monthly number of CIED implantation increased by 15.60% (95% CI: 14.34–16.85%, *p* < 0.001) compared with 2020. For every 10-fold increase in the number of COVID-19 cases, the monthly number of pacemaker implantation decreased by 429 in 2021, while it decreased by 676 in 2020. The proportion of CIED implantation in secondary medical centers increased from 52.84% in 2019 to 56.77% in 2021 (*p* < 0.001). For every 10-fold increase in regional accumulated COVID-19 cases, the proportion of CIED implantation in secondary centers increased by 6.43% (95% CI: 0.47–12.39%, *p* = 0.036).

**Conclusion:**

The impact of the COVID-19 pandemic on the number of CIED implantation is diminishing in China. Improving the ability of secondary medical centers to undertake more operations may be a critical way to relieve the strain on healthcare resources during the epidemic.

## Introduction

Coronavirus disease 2019 (COVID-19) has caused unprecedented strains on healthcare resources (1, 2), with over 500 million infections and six million deaths caused by the illness over the past two years (3). In response, governments have imposed restrictions on movement and social distancing to mitigate the spread (4–6). Additionally, cardiology societies have issued guidelines recommending the postponement or cancellation of any non-urgent elective procedures to conserve healthcare resources, including the placement of cardiac implantable electronic devices (CIED) (7). Focusing on the early stages of the outbreak, studies from several countries have reported a substantial reduction in the number of cardiac interventions (8–16). However, the survival benefits of CIED implantation have been clearly established (17), and delayed implantation may lead to poor patient outcomes. After the first pandemic wave, several critical measures that have been adopted in many countries, such as the development of COVID-19 vaccines (18–21) and the application of telemedicine (22–24), may have had a significant impact on the spread of the disease and the management of cardiac interventional procedures. But no studies have yet reported changes in CIED implantation during the following pandemic.

As the first country to report COVID-19, China has also faced an unprecedented public health challenge. Studies have reported decreases in coronary interventional surgery in Hubei (5) and Beijing (15). Focused on arrhythmia, the Chinese Society of Arrhythmia (CSA) and the Chinese society of pacing and electrophysiology (CSPE) have co-proposed “3R telemedicine project” in 2020, aiming to improve the ability of secondary centers in performing interventional procedures. However, no studies have examined the status of CIED implantation in China. This study aimed to assess the impact of COVID-19 on CIED implantation in terms of quantity and structure in China during various periods, and provide experience in the management of CIED implantation during the pandemic.

## Methods

### The national center for cardiovascular quality improvement database

We retrospectively analyzed archived data from the National Center for Cardiovascular Quality Improvement (NCCQI) database. NCCQI was established by the National Center for Cardiovascular Diseases under the guidance of the National Health Commission of the People's republic of China, to strengthen the quality management of cardiovascular diseases. All data were reviewed and confirmed by the provincial sub-centers and then aggregated to the National Center for Cardiovascular Diseases and managed by the NCCQI committee. The number and types of newly CIED implantation (including pacemaker [PM], implantable cardiac defibrillator [ICD], and cardiac resynchronization therapy [CRT]) were uploaded to the database every month in each hospital. By December 2021, 1227 hospitals that have the facility and ability to perform CIED implantation from 31 provinces in China (all except Macau, Hong Kong, and Taiwan) had participated. All data used in this article are verifiable and have been approved by the committee.

### The COVID-19 pandemic in China

**Figure 1:**
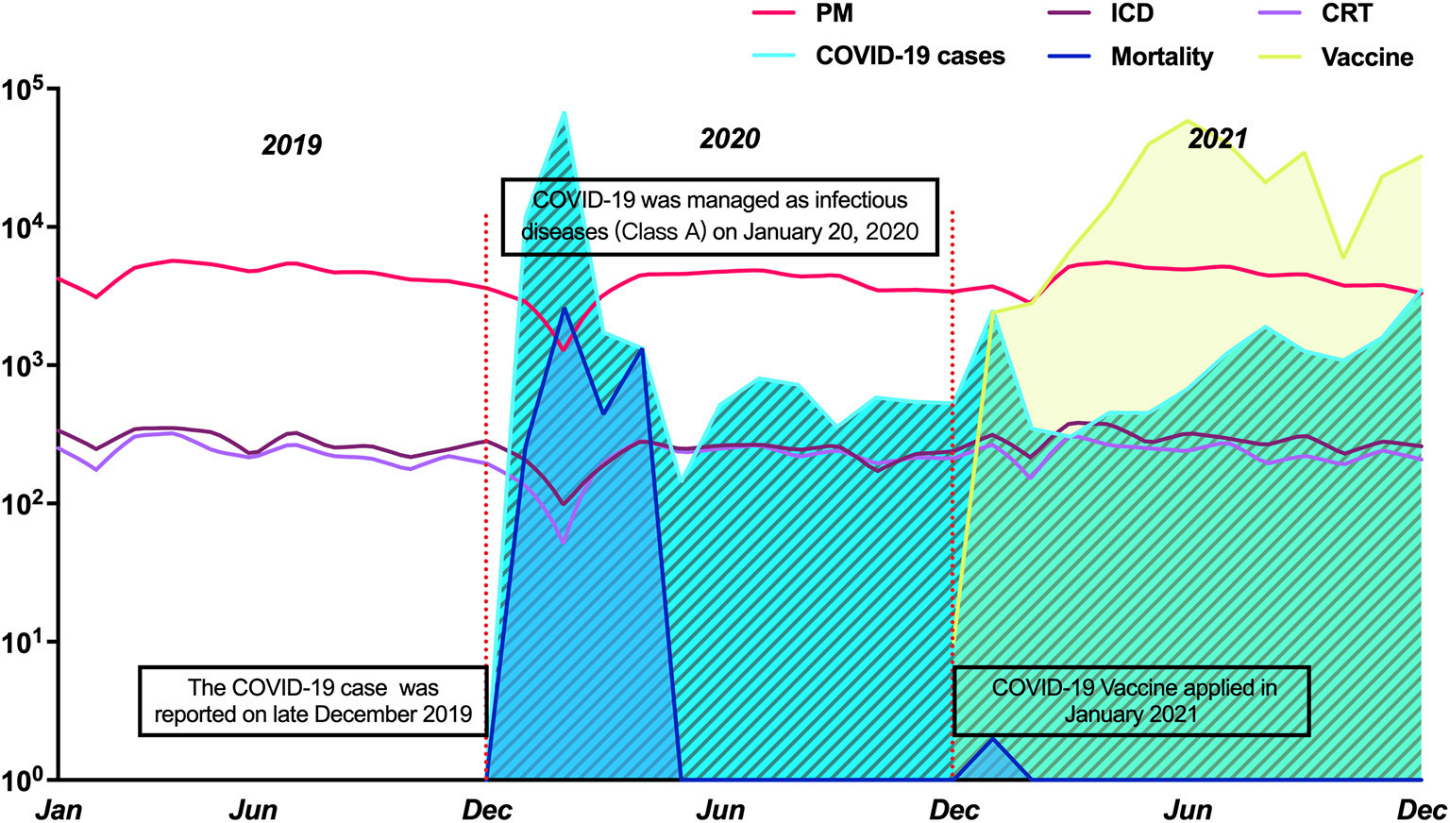
The trend of CIED implantation from 2019 to 2021 and COVID-19 pandemic in China. COVID-19: Coronavirus disease 2019; CRT, cardiac resynchronization therapy; ICD, implantable cardiac defibrillator; PM, pacemaker.

In China, COVID-19 was managed as a Class A (highest level) infectious disease in January 2020 (Figure 1) after COVID-19 cases were reported in late December 2019. By December 31, 2021, a total of 102,341 cases had been confirmed in 31 provinces (all except Macau, Hong Kong, and Taiwan). The COVID-19 vaccine began being applied in January 2021, and over 2,835 million COVID-19 vaccination doses have been administered in total by December 31, 2021. All data and information on the COVID-19 pandemic in China mentioned in this article were collected from the websites of the National Health Commission (http://www.nhc.gov.cn/wjw/index.shtml) and provincial health commissions. The visualized data are also available from the website (https://2019ncov.chinacdc.cn/2019-nCoV/) established by National Centers for Disease Control.

### Data collection in different periods of COVID-19 in China

Data about newly confirmed and cumulative COVID-19 cases every month were collected across 31 provinces, as well as the monthly COVID-19-related deaths and the number of vaccinations administered. The year 2019 was defined as the pre-COVID-19 period, and data on CIED implantations were used as the control group. Data from 2020 and 2021 were used to compare the annual changes in CIED implantations in the early and the following stage of the pandemic, respectively. Data on monthly or regional implantations of different types of devices nationwide were collected to compare the differences during the pre-COVID-19 period, 2020, and 2021. All data used were mutually checked by four authors and finally reviewed by the corresponding author. The requirement for written informed consent was waived owing to the retrospective study design and results without patient characteristics.

### The classification of centers and the 3R telemedicine project

In response to challenges proposed by the pandemic, CSA and CSPE co-proposed the “3R Telemedicine Project” in 2020, aiming to improve the ability of secondary centers to manage arrhythmia interventions. Specifically, this project directs the major centers of each province to deliver remote training to secondary centers, providing real-time guidance for operations, with postoperative remote follow-up management. To compare the changes in CIED implantation proportion of different centers, hospitals in the NCCQI were divided into major centers and secondary centers. The major centers were the top three hospitals for CIED implantation, and the hospitals with fewer CIED implants in each province were defined as secondary centers. Of the 31 total provinces, three were excluded because there were fewer than six centers; thus, 1,218 hospitals from 28 provinces were included in the analysis.

### Statistical analysis

Continuous variables are summarized as median (interquartile range) for skewed distributions. Categorical variables, presented as numbers with relative percentages, were compared using the chi-squared test. The Bonferonni procedure was used to adjust for multiple testing. Assuming that the monthly number of patients followed a Poisson distribution with a log link function, generalized linear models were established to determine the difference in monthly CIED implantations between different periods. The change in monthly CIED implantations between different periods was expressed as the change in the rate of events. The data about COVID-19 cases were logarithmically transformed (Log10). Scatter diagrams and simple linear regression models were used to assess the relationship between changes in CIED implantation and monthly regional confirmed COVID-19 cases. The relationship between changes in the CIED proportion in secondary hospitals and regional COVID-19 cases was also assessed by simple linear regression models. Statistical significance was set at *P* < 0.05, and all tests were two-sided. Statistical analyses were conducted using R version 4.0.3 (The R Foundation for Statistical Computing, Vienna, Austria) and SPSS statistical software version 23 (IBM Corp., Armonk, N.Y., USA).

## Results

### Overall CIED implantations and the COVID-19 pandemic

A total of 177,263 patients who had undergone CIED implantation from 1,227 hospitals in 31 provinces in China between January 01, 2019, and December 31, 2021, were included in the analysis. Figure 1 shows the trends of various types of CIED implantation and the corresponding status of the COVID-19 pandemic. The monthly numbers of CIED implantations in the three periods and corresponding COVID-19 cases are shown in Table 1. The total number of CIED implantations decreased from 63,877 (PM: 89.57%; ICD:5.78%; CRT:4.66%) in the pre-COVID-19 period to 52591 (PM,89.66%; ICD, 6.04%; CRT,4.93%) in 2020. One year later, the number had increased to 60795 (PM:89.10%; ICD:6.11%; CRT:4.79%) in 2021.

**Table 1:** The monthly number of CIED implantation and confirmed COVID-19 cases from 2019 to 2021.

**Month**	**2019**		**2020**			**2021**		
	**CIED number**	**CIED number**	**Changes (vs. 2019), %**	**COVID-19 cases**	**CIED number**	**Changes (vs. 2019),%**	**Changes (vs. 2020), %**	**COVID-19 cases**
January	5,010	3,341	−33.31%	11,791	4,359	−12.99%	30.47%	2,493
February	3,625	1,396	−61.49%	68,033	3,236	−10.73%	131.81%	348
March	6,009	3,678	−38.79%	1,730	6,208	3.31%	68.79%	305
April	6,679	5,246	−21.46%	1,320	6,483	−2.93%	23.58%	454
May	6,138	5,215	−15.04%	143	5,808	−5.38%	11.37%	451
June	5,375	5,461	1.60%	517	5,743	6.85%	5.16%	670
July	6,383	5,623	−11.91%	803	5,895	−7.65%	4.84%	1,213
August	5,403	5,020	−7.09%	721	4,989	−7.66%	−0.62%	1,893
September	5,395	5,260	−2.50%	356	5,220	−3.24%	−0.76%	1,264
October	4,788	4,014	−16.17%	583	4,479	−6.45%	11.58%	1,081
November	4,772	4,207	−11.84%	4	4,463	−6.48%	6.09%	1,581
December	4,300	4,130	−3.95%	529	3,912	−9.02%	−5.28%	3,490
Total	63,877	52,591	−17.67%	87,071	60,795	−4.82%	15.60%	15,243

CIED, cardiac implantable electronic device; COVID-19, Coronavirus disease 2019.

### Monthly changes in CIED implantations in different periods

**Figure 2:**
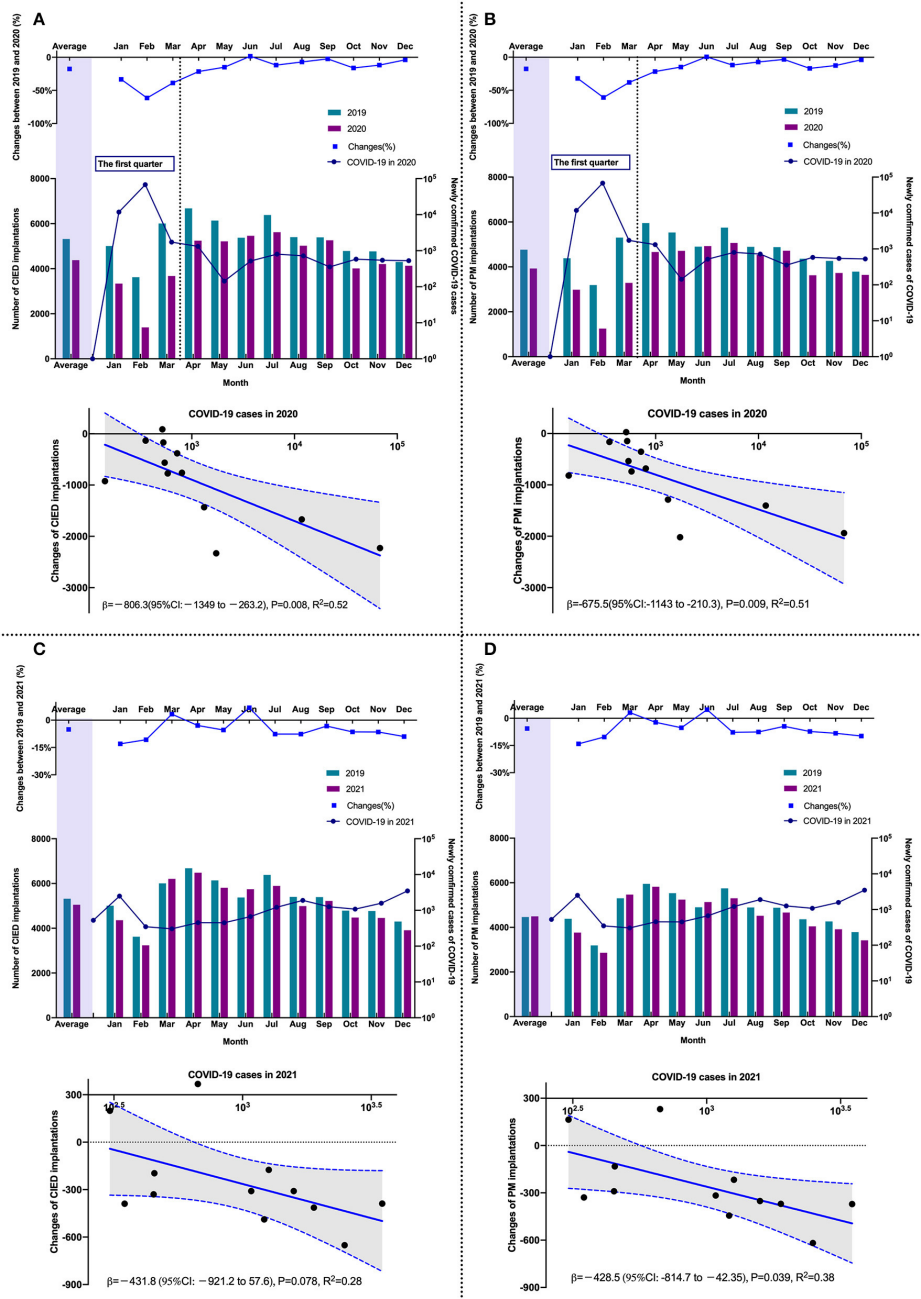
The comparison of the monthly number of CIED in different periods and the relationship between changes and COVID-19 cases. **(A)** CIED (2019 vs. 2020); **(B)** PM (2019 vs. 2020); **(C)** CIED (2019 vs. 2021); **(D)** PM (2019 vs. 2021). CIED, cardiac implantable electronic device; COVID-19, Coronavirus disease 2019; PM, pacemaker.

The monthly numbers of CIED implantations and confirmed COVID-19 cases in 2020 are shown in Figure 2A. Compared with the pre-COVID-19 period, monthly CIED implantations decreased by 17.67% (95% CI: 16.62–18.72%, *p* < 0.001) in 2020. In the scatter plot, there was an inverse linear relationship between changes in CIED implantation and monthly confirmed COVID-19 cases (β = −806; 95% CI: −1,349–−263, *P* = 0.008) (Figure 2A). The monthly number of PM implantation decreased significantly by 17.58% (95% CI: 16.48–18.69%, *p* < 0.001), with an inverse linear relationship between changes in PM and confirmed cases (β = −676; 95% CI: −1,143–−210, *P* = 0.009) (Figure 2B), indicating that the monthly number of PM implantations decreased 676 per 10-fold increase in the monthly number of COVID-19 cases. The number of ICD and CRT implantation decreased by 22.82% (95% CI: 18.53–27.21%, *p* < 0.001) and 12.90% (95% CI: 7.99–17.82%, *p* < 0.001), respectively (Supplementary Figure S1). Additionally, the first quarter of 2020 was the most pronounced period for CIED reductions (Figure 1). The total number of CIED implantation decreased by 6,229 compared to the same period in 2019 and accounted for 55.2% of the total decline in 2020. Also, during this period, there were 81,554 cases confirmed and 3,312 COVID-19-related deaths reported, accounting for 93.7% of confirmed cases and 71.5% of deaths for the entire 2020 year, respectively. Since then, the monthly number of COVID-19 cases and associated deaths have decreased significantly and held steady, while similarly, the magnitude of change in CIED implantation has kept stable.

As shown in Figure 2C, compared with the pre-COVID-19 period, the number of CIED implantations per month decreased by 4.82% (95% CI: 3.74–5.91%, *p* < 0.001) in 2021, following the decline in PM implantations (decreased by 5.32%; 95% CI: 4.18–6.47%, *p* < 0.001) (Figure 2D). An inverse linear relationship was observed between monthly changes in PM implantations and the corresponding COVID-19 cases (β = −429; 95% CI: −815–−42, *p* = 0.033) (Figure 2D), indicating that for every 10-fold increase in case number per month, the monthly number of PM implantations decreased by 429 in 2021, while it decreased by 676 in 2020. The decreased number in 2020 was 36.5% higher than in 2021. The monthly numbers of ICD (0.68%; 95% CI: −3.89–5.25%, *p* = 0.771) and CRT (−2.08%; 95%CI: −7.14–2.97%, *p* = 0.419) implantations were similar to those in the pre-COVID-19 period (Supplementary Figure S1).

Compared with 87,071 COVID-19 cases in 2020, a total of 15,243 cases were reported in 2021. The monthly number of CIED implantations increased by 15.60% (95% CI: 14.34–16.85%, *p* < 0.001) in 2021. The monthly number of patients who underwent PM implantation increased by 14.88% (95% CI: 13.55–16.20, *p* < 0.001), whereas ICD implantation increased by 30.45% (95% CI: 24.88–36.03%, *p* < 0.001) and CRT implantation increased by 12.42% (95% CI: 6.81–18.03%, *p* < 0.001). In particular, the total number of PM and ICD implantations increased by 62.2 and 88.3%, respectively, in the first quarter of 2021 compared to 2020 (Supplementary Figure S2).

### Regional distribution of CIED implantation

**Figure 3:**
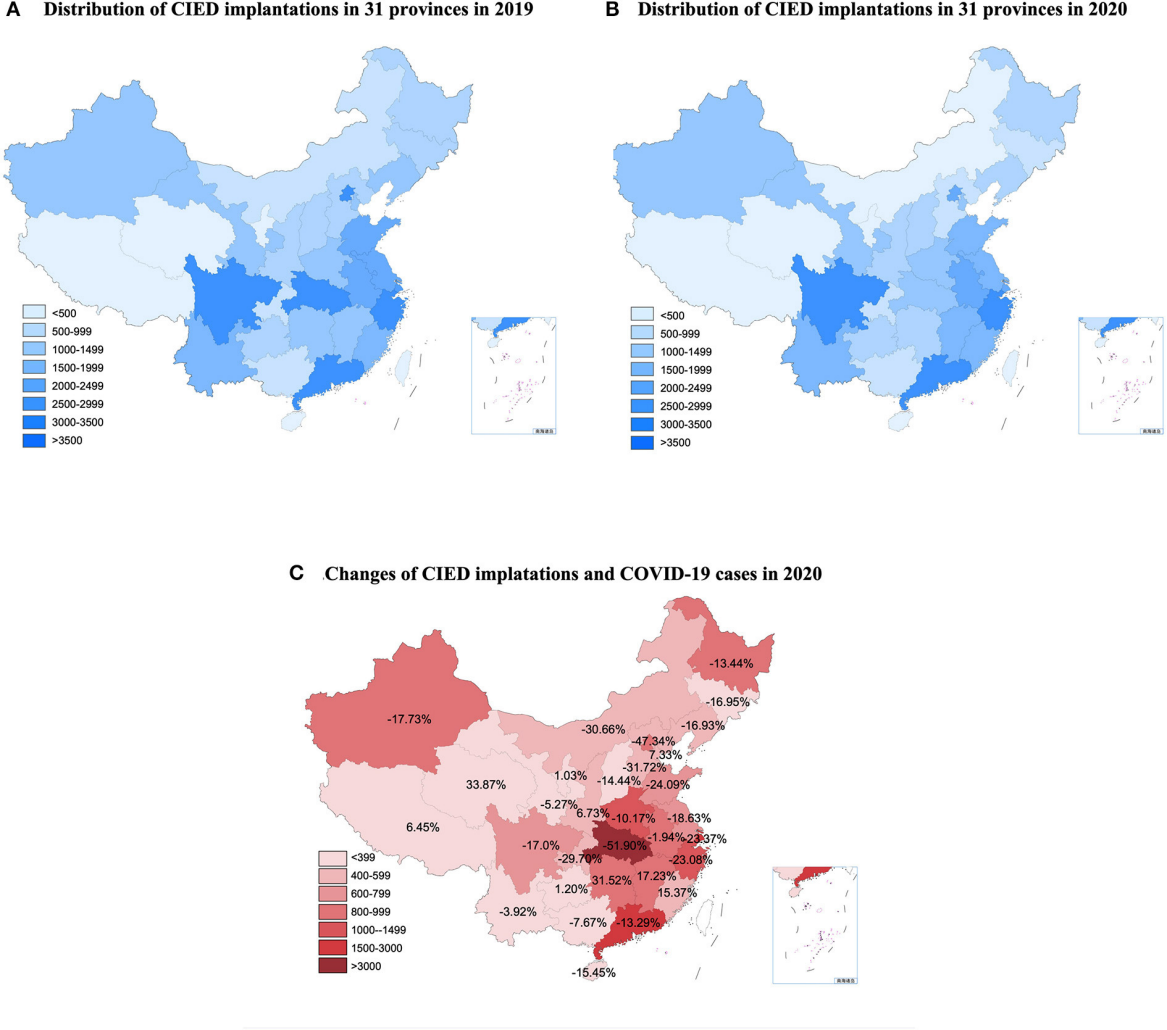
The distribution of CIED implantations in the pre-COVID period and 2020 and the relationship between changes and COVID-19 cases. CIED, cardiac implantable electronic device; COVID-19, Coronavirus disease 2019. **(A)** Distribution of CIED implantations in 31 provinces in 2019. **(B)** Distribution of CIED implantations in 31 provinces in 2020. **(C)** Changes of CIED implantations and COVID-19 cases in 2020.

The regional distribution of CIED implantation in the pre-COVID-19 period and 2020 is shown in Figures 3A,B. The number of CIED implantation changed between 2019 and 2020, and the corresponding confirmed COVID-19 cases for 2020 are shown in Figure 3C. Among included 31 provinces, 24 showed a decrease in CIED implantation in 2020, from −1.94–−51.9%, with 19 provinces decreasing by more than 10% and 10 provinces decreasing by more than 20%. Hubei Province, the initial outbreak area of COVID-19 in China, experienced a 51.90% decrease in the number of CIED implantation in 2020. There was a significant inverse linear relationship between changes in CIED implantation and regional COVID-19 cases with a β of −13.68 (95% CI: −21.19–−6.16, *p* < 0.001). It indicates that for every 10-fold increase in the regional number of cases, the number of CIED implantation decreased by 13.68%. A similar linear relationship could be also observed in PM (β = −13.39; 95% CI: −20.78–−6.01, *p* < 0.001) and ICD (β = −17.95; 95% CI: −34.61–−1.29, *p* = 0.036) groups. In 2021, 22 provinces showed an increase in total CIED implantation, with 17 provinces increasing by more than 10% and 14 provinces increasing by more than 20%. In particular, 16 provinces have already recovered or exceeded their CIED implantation volumes in the pre-COVID-19 period, ranging from 4.27 to 63.53%. However, the linear relationship between changes in CIED implantation and regional COVID-19 cases was not significant anymore in 2021 (β = −12.40; 95% CI: −26.77–−1.97, *p* = 0.088).

### Changes in CIED proportion in different levels of centers

As shown in Table 2, from 2019 to 2021, all 84 major centers from 28 provinces were responsible for 45.79% of CIED implantation; however, the composition ratio gradually changed. Compared with 52.49% in the pre-COVID-19 period, the proportion of CIED implantation in secondary centers increased to 54.15% in 2020 (*p* < 0.001) and further increased to 56.77% by 2021 (*p* < 0.001). This followed the increasing proportion of PM implantations in secondary centers, which increased to 56.35% in 2020 (*p* < 0.001) and 59.03% in 2021 (*p* < 0.001). Although major centers were responsible for more ICD implantation, the ICD implantation proportion in secondary centers increased by 5.7% from 2019 to 2021 (*p* < 0.001). However, there were no significant changes in the proportion of CRT implantation over the three years (*p* = 0.742), and major centers accounted for two-thirds of the CRT implantation.

**Table 2:** Changes in CIED implantation in major and secondary centers from 2019 to 2021.

	**Major center (*****n*** = **84)**		**Secondary center (*****n*** = **1,134)**		**Total**	***P* value**
	**Number**	**Percentage**	**Number**	**Percentage**		
**CIED**						
2019	29,902	47.16%	33,510	52.84%	63,412	<0.001
2020	23,861	45.85%	28,185	54.15%	52,046	
2021	26,030	43.23%	34,182	56.77%	60,212	
Total	79,793	45.42%	95,877	54.58%	175,670	
**PM**						
2019	25,618	45.13%	31,144	54.87%	56,762	<0.001
2020	20,353	43.65%	26,270	56.35%	46,623	
2021	21,961	40.97%	31,642	59.03%	53,603	
Total	67,932	43.27%	89,056	56.73%	156,988	
**ICD**						
2019	2,252	61.23%	1,426	38.77%	3,678	<0.001
2020	1,736	61.19%	1,101	38.81%	2,837	
2021	2,060	55.56%	1,648	44.44%	3,708	
Total	6,048	59.16%	4,175	40.84%	10,223	
**CRT**						
2019	2,032	68.37%	940	31.63%	2,972	0.742
2020	1,772	68.52%	814	31.48%	2,586	
2021	2,009	69.25%	892	30.75%	2,901	
Total	5,813	68.72%	2,646	31.28%	8,459	

CIED, cardiac implantable electronic device; CRT, cardiac resynchronization therapy; ICD, implantable cardiac defibrillator; PM, pacemaker.

The results of all pairwise comparisons are significant in CIED and PM groups (all Bonferroni-adjusted *P* < 0.001), but not in the CRT group (*P* > 0.05). For ICD group, no difference between 2019 and 2020 (*P* = 0.995), while there was a significant increase in 2021 (Bonferroni-adjusted *P* < 0.001).

**Figure 4:**
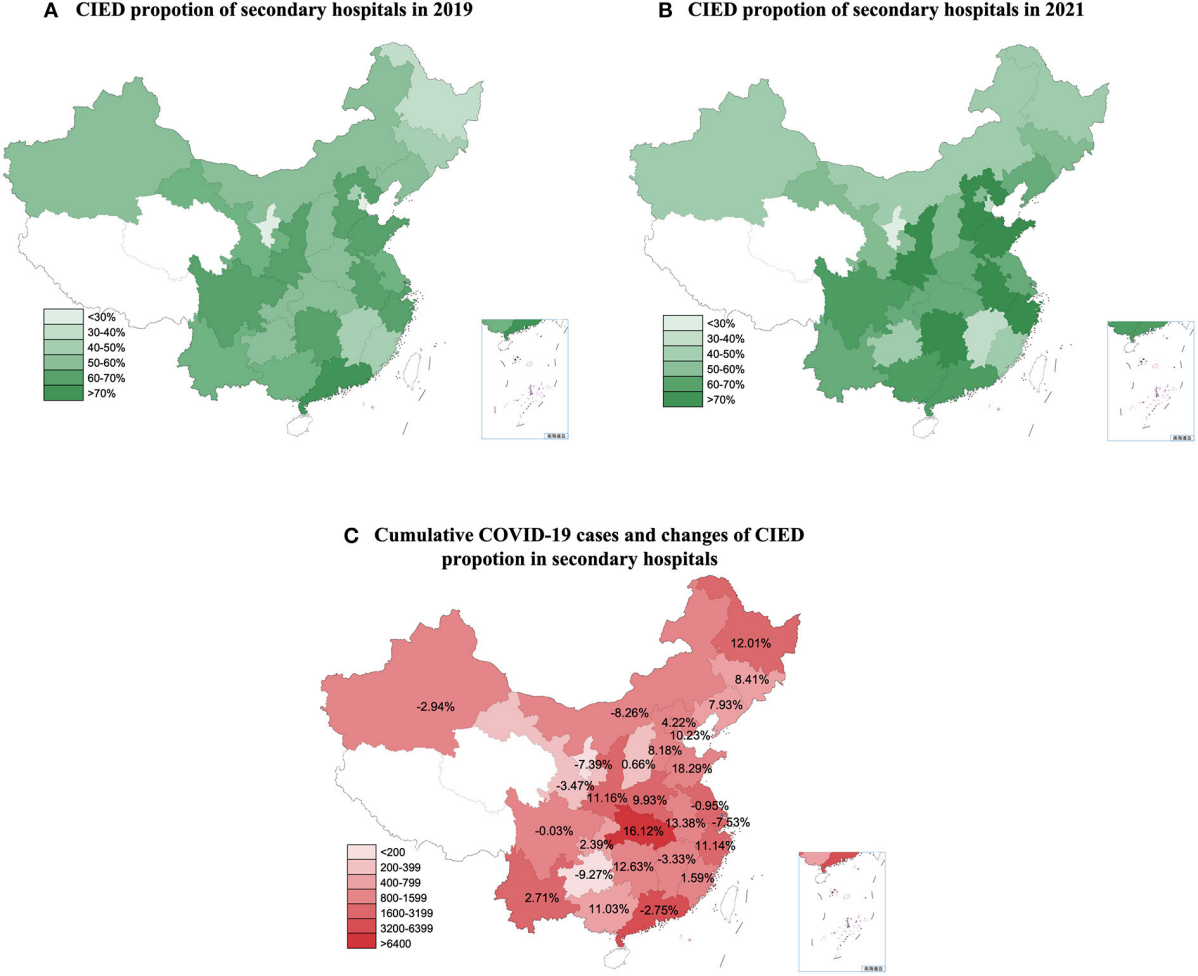
The proportion of CIED implantations in secondary centers in 2019 and 2021 and the relationship between the changes and cumulative COVID-19 cases. CIED, cardiac implantable electronic device. COVID-19, Coronavirus disease 2019. **(A)** CIED proportion of secondary hospitals in 2019. **(B)** CIED proportion of secondary hospitals in 2021. **(C)** Cumulative COVID-19 cases and changes CIED proportion of secondary hospitals.

The proportion of CIED implantation in secondary centers in each province during the pre-COVID period and 2021 are shown in Figures 4A,B. A linear relationship was observed between changes in the proportion of CIED implantations in regional secondary centers and the number of accumulated COVID-19 cases in each province (β = 6.43 [95% CI: 0.47–12.39], *p* = 0.036) (Figure 4C). This indicated that the proportion of CIED implantations in secondary centers increased by 6.43% per 10-fold increase in the regionally accumulated COVID-19 cases. The average increases in the proportion of PM and ICD implantations in regional secondary hospitals were 4.29% (95% CI: 1.07–7.52%) and 9.37% (95% CI: 4.93–13.82%), respectively. A linear relationship was observed between changes in the proportion of PM implantations in regional secondary centers and the corresponding number of accumulated COVID-19 cases (β = 7.39 [95% CI: 1.28–13.49], *P* = 0.019).

## Discussion

This was the first report on the nationwide status of CIED implantation in China comparing CIED implantation changes in quantity and structure from 2019 to 2021. The main findings were as follows: (1) Compared with the pre-COVID-19 period, the monthly numbers of CIED implantation decreased significantly in 2020, which was closely related to the severity of the epidemic; (2) the number of CIED implantation increased significantly in 2021, and secondary centers were able to afford more proportion of PM and ICD implantations during the COVID-19 pandemic.

During the early phase of the COVID-19 pandemic, CIED implantations decreased by 56.5% in northeastern Spain (9), 39.38% in Poland (12), 28% in the Veneto region of Italy (10), and 48% in northwestern Greece (13). In agreement with these reports, the number of CIED implantations in China also decreased significantly in 2020. Possible reasons for the decline in the early stages have been well-discussed (9–13). First, due to a lack of experience in managing COVID-19, the sudden outbreak of a large number of cases has caused unprecedented strains on healthcare resources, which is supported by the significantly higher number of related deaths. Elective procedures and non-critical visits may be postponed or canceled to protect hospital resources. Consequently, the reduction in CIED implantation in China is most pronounced at this time. Moreover, governments implemented large-scale public health measures such as stay-at-home orders and lockdowns, resulting in a decline in routine medical consultations. Notably, a reduction in urgent PM implantation was reported in Italy after the COVID-19 outbreak (11). One possible explanation is that many patients, fearing infection, may not seek medical services even for severe symptoms. Therefore, it is essential to help patients properly assess arrhythmia-related symptoms in order to reduce serious complications and even death, resulting from delayed treatment.

In China, as compared with the reduction of 17.9% of CIED implantation in 2020, the previous study reported that the number of patients who received percutaneous coronary intervention decreased by 12% during the initial outbreak of COVID-19 (15). In the UK, the reduction of CIED was also more pronounced than percutaneous coronary intervention, but lower than the volume of cardiac ablation (9). It suggests that the volume of all types of cardiac interventions has declined to vary degrees due to the COVID-19 pandemic. Patients with bradycardia seem to be more restricted in terms of interventional therapy compared to patients with coronary artery disease.

Unlike in the early stages of the pandemic, the number of CIED implantations in 2021 increased significantly. Moreover, compared with the pe-COVID-19 period, an inverse linear relationship was observed between monthly changes in PM implantations and corresponding monthly COVID-19 cases in 2020 and 2021. However, for every ten-fold increase in the monthly number of cases, the monthly PM implantations decreased by 429 in 2021 but decreased by 36.5% from 676 in 2020. This indicates that institutions were able to afford more operations in 2021 when faced with the COVID-19 pandemic. It suggests that the impact of the COVID-19 pandemic on CIED implantation is diminishing in China. A reasonable reason may be that the pandemic has been effectively controlled to a certain extent, supported by the significantly reduction in newly confirmed COVID-19 cases in 2021. Thus the direct occupation of healthcare resources by COVID-19 may be significantly alleviated.

Although the volume of CIED implantations increased significantly in 2021, further analysis indicated that the growth was mainly attributed to the increase in implantation in secondary centers. The proportion of CIED implantation in secondary centers increased from 52.84% in the pre-COVID-19 period to 56.77% in 2021. Moreover, for every 10-fold increase in regional COVID-19 cases, the CIED implantation proportion of secondary centers increased by 6.43% in 2021. This indicates that in provinces with more experience with fighting COVID-19, regional secondary centers were able to undertake more operations. A possible reason for this is that the major centers were primarily located in central cities with a good flow of people, which were more likely to be constrained by strict policies. Therefore, patients are unable to travel to major centers for surgery and may have to visit local secondary hospitals. Under these circumstances, the “3R telemedicine project” may have contributed to the changes. To better improve the ability of secondary medical centers in CIED implantation, the following areas deserve more attention. First, to establish a timely and stable connection between major regional medical centers and surrounding secondary hospitals in order to provide timely and effective medical services to patients. Second, Amid the pandemic, telemedicine helped ensure the continuity of medical care (22). Remote-monitored CIED has also been recommended to reduce the need for non-urgent clinic visits (7, 24). Thus, more systematic telemedicine, including remote training and real-time guidance, and remote follow-up may be recommended to realize the full potential of secondary centers. Finally, major centers have accounted for two-thirds of CRT implantation over the 3 years. Considering the safety and success rate of the procedure, routine PM and selected ICD procedures may be performed in secondary hospitals, especially in those with relevant experience. But for CRT candidates with refractory heart failure, a medical visit to a major center is still recommended.

## Limitations

This study had several limitations. First, it was based on an administrative database rather than a clinical database. While this approach was useful for collecting national data, it was scant in clinical detail such as patient characteristics. Therefore, more baseline characteristics of patients deserve further identification, such as the urgency of the condition and the severity of comorbidities. Additionally, there may be a few hospitals with CIED implantation capacity that are not currently included in the NCCQI system, which may lead to an underestimation of the overall implantation volume. Second, this study was observational in nature. Although certain trends have been observed within different pandemic periods, we cannot conclude that the reported associations were causative. Both the change in volume and structure may have been affected by many factors, and a single factor or intervention may have only partially influenced the outcome. Third, telemedicine was proposed to improve the ability of secondary centers to undertake CIED implantation. Although the proportion of CIED implantation in secondary centers increased significantly during the pandemic, the effect of telemedicine has not been quantified. More detailed research is needed to verify the safety and effectiveness of telemedicine in CIED implantation.

## Conclusion

Compared with the substantial reduction in the number of CIED implantation in 2020, CIED implantations increased significantly in 2021. The impact of the COVID-19 pandemic on CIED implantation is diminishing in China. Secondary centers have great potential to undertake more routine operations under systematic guidance, well worth our deep concern. Improving the ability of secondary centers may be a critical way to relieve the strain on healthcare resources during the epidemic.

## Data availability statement

The raw data supporting the conclusions of this article will be made available by the authors, without undue reservation.

## Author contributions

C-dC and SZhao had the idea for the study and contributed to the study design. C-dC, JJ, SZhao, and X-hN collected the data, conducted a literature review, and drafted the manuscript. X-hN and SZhan provided guidance, support for statistical analysis, and provided support for the development of the NCQQI platform. NL and PL interpreted data and coded the figures. All authors contributed to the study design, literature review, data analysis, manuscript writing, revision, read, and approved the final manuscript.

## Funding

This work was supported by the Natural Science Foundation of China (81470466) and the National Science and Technology Pillar Program during the 12th Five-Year Plan Period (2011BAI11B02).

## Conflict of interest

The authors declare that the research was conducted in the absence of any commercial or financial relationships that could be construed as a potential conflict of interest.

## Publisher's note

All claims expressed in this article are solely those of the authors and do not necessarily represent those of their affiliated organizations, or those of the publisher, the editors and the reviewers. Any product that may be evaluated in this article, or claim that may be made by its manufacturer, is not guaranteed or endorsed by the publisher.

## References

[1] Miller IF Becker AD Grenfell BT Metcalf JE. Disease and healthcare burden of COVID-19 in the United States. Nat Med. (2020) 26:1212–7. 10.1038/s41591-020-0952-y 32546823

[2] Moynihan R Johansson M Maybee A Lang E Legare F. COVID-19: an opportunity to reduce unnecessary healthcare. BMJ. (2020) 370:m2752. 10.1136/bmj.m2752 32665257

[3] World Health Organization. Coronavirus disease (COVID-19) situation reports. (2021). Available online at: https://www.who.int/emergencies/diseases/novel-coronavirus-2019/situation-reports/ (accessed May 05, 2021).

[4] Yamaji K Kohsaka S Inohara T Numasawa Y Ando H Wada H. Percutaneous coronary intervention during the COVID-19 pandemic in Japan: insights from the nationwide registration data. Lancet Reg Health West Pac. (2022) 22:100434. 10.1016/j.lanwpc.2022.100434 35330940 PMC8939342

[5] Mafham MM Spata E Goldacre R Gair D Curnow P Bray M. COVID-19 pandemic and admission rates for and management of acute coronary syndromes in England. Lancet. (2020) 396:381–9. 10.1016/S0140-6736(20)31356-8 32679111 PMC7429983

[6] Xiang D Xiang X Zhang W Yi S Zhang J Gu X. Management and Outcomes of Patients With STEMI During the COVID-19 Pandemic in China. J Am Coll Cardiol. (2020) 76:1318–24. 10.1016/j.jacc.2020.06.039 32828614 PMC7438071

[7] Lakkireddy DR Chung MK Gopinathannair R Patton KK Gluckman TJ Turagam M. Guidance for cardiac electrophysiology during the coronavirus (COVID-19) pandemic from the Heart Rhythm Society COVID-19 task force; Electrophysiology Section of the American College of Cardiology; and the Electrocardiography and Arrhythmias Committee of the Council on Clinical Cardiology, American Heart Association. Heart Rhythm. (2020) 17:e233–41. 10.1016/j.hrthm.2020.03.028 32247013 PMC7118697

[8] Garcia S Albaghdadi MS Meraj PM Schmidt C Garberich R Jaffer FA. Reduction in ST-segment elevation cardiac catheterization laboratory activations in the United States during COVID-19 pandemic. J Am Coll Cardiol. (2020) 75:2871–2. 10.1016/j.jacc.2020.04.011 32283124 PMC7151384

[9] Leyva F Zegard A Okafor O Stegemann B Ludman P Qiu T. Cardiac operations and interventions during the COVID-19 pandemic: a nationwide perspective. Europace. (2021) 23:928–36. 10.1093/europace/euab013 33778881 PMC8083650

[10] Arbelo E Angera I Trucco E Rivas-Gándara N Guerra JM Bisbal F. Reduction in new cardiac electronic device implantations in Catalonia during COVID-19. Europace. (2021) 23:456–63. 10.1093/europace/euab011 33595062 PMC7928966

[11] Migliore F Zorzi A Gregori D Del Monte A Falzone PV Verlato R. Urgent pacemaker implantation rates in the Veneto region of Italy after the COVID-19 outbreak. Circ Arrhythm Electrophysiol. (2020) 13:e008722. 10.1161/CIRCEP.120.008722 32434373 PMC7299094

[12] Schwab JO Wiese J Hauser T. The influence of the 2020 COVID-19 pandemic on the implantation rates of cardiac implantable electronic devices in Germany: changes between 2020 Q1-Q3 and 2019 Q1-Q3. Eur Heart J Qual Care Clin Outcomes. (2022) 8:104–12. 10.1093/ehjqcco/qcab091 34849668 PMC8690261

[13] Filipecki A Orszulak M Tajstra M Kowalski O Skrzypek M Kalarus Z. Cardiac implantable electronic devices procedures and their recipients characteristic during COVID-19 pandemic: 38 million population analysis. Cardiol J. (2022) 29:27–32. 10.5603/CJ.a2021.0170 34931693 PMC8890427

[14] Bechlioulis A Sfairopoulos D Korantzopoulos P. Impact of COVID-19 pandemic on cardiac electronic device implantations in Northwestern Greece. Am J Cardiovasc Dis. (2021) 11:489–93. 34548948 PMC8449197

[15] He L Lu F Du X Long D Sang C Tang R. Impact of COVID-19 pandemic on hospital admissions of acute coronary syndrome: a Beijing Inpatient database study. Lancet Reg Health West Pac. (2022) 19:100335. 10.1016/j.lanwpc.2021.100335 34927111 PMC8665660

[16] Li J Zhang N Zhou Z Huang X Fang W Yan H. Twin peaks of in-hospital mortality among patients with STEMI across five phases of COVID-19 outbreak in China: a nation-wide study. Sci China Life Sci. (2022) 65:1855–65. 10.1007/s11427-021-2046-4 35524908 PMC9077341

[17] Glikson M Nielsen JC Kronborg MB Michowitz Y Auricchio A Barbash IS. 2021 ESC Guidelines on cardiac pacing and cardiac resynchronization therapy. Eur Heart J. (2021) 42:3427–520. 10.1093/eurheartj/ehab364 34586378

[18] Tregoning JS Flight KE Higham SL Wang Z Pierce B. Progress of the COVID-19 vaccine effort: viruses, vaccines and variants versus efficacy, effectiveness and escape. Nat Rev Immunol. (2021) 21:626–36. 10.1038/s41577-021-00592-1 34373623 PMC8351583

[19] Thomas SJ Moreira ED Jr Kitchin N Absalon J Gurtman A Lockhart S. Safety and Efficacy of the BNT162b2 mRNA Covid-19 Vaccine through 6 Months. N Engl J Med. (2021) 385:1761–73. 10.1056/NEJMoa2110345 34525277 PMC8461570

[20] Feikin DR Higdon MM Abu-Raddad LJ Andrews N Araos R Goldberg Y. Duration of effectiveness of vaccines against SARS-CoV-2 infection and COVID-19 disease: results of a systematic review and meta-regression. Lancet. (2022) 399:924–44. 10.1016/S0140-6736(22)00152-0 35202601 PMC8863502

[21] Jung J Kim JY Park H Park S Lim JS Lim SY. Transmission and Infectious SARS-CoV-2 Shedding Kinetics in Vaccinated and Unvaccinated Individuals. JAMA Netw Open. (2022) 5:e2213606. 10.1001/jamanetworkopen.2022.13606 35608859 PMC9131744

[22] Pogorzelska K Chlabicz S. Patient satisfaction with telemedicine during the COVID-19 pandemic-a systematic review. Int J Environ Res Public Health. (2022) 19:6113. 10.3390/ijerph19106113 35627650 PMC9140408

[23] Tong L Xiong S Hou J Li J Qin S Zhang Y. Cloud follow-up in patients with cardiovascular implantable electronic devices: a single-region study in China. Front Cardiovasc Med. (2022) 9:864398. 10.3389/fcvm.2022.864398 35615564 PMC9124837

[24] Maines M Palmisano P Del Greco M Melissano D De Bonis S Baccillieri S. Impact of COVID-19 Pandemic on Remote Monitoring of Cardiac Implantable Electronic Devices in Italy: Results of a Survey Promoted by AIAC (Italian Association of Arrhythmology and Cardiac Pacing). J Clin Med. (2021) 10:4086. 10.3390/jcm10184086 34575197 PMC8469719

