# Supplementary material for: Impact of the COVID-19 pandemic on cardiac implantable electronic device implantation in China: Insights from 2 years of changing pandemic conditions
Source: Front Public Health. 2022 Nov 22;10:1031241. doi: 10.3389/fpubh.2022.1031241 (PMC9723342; doi:10.3389/fpubh.2022.1031241)
Supplement: Supplementary file 1 [file Table_1.DOCX]

Supplementary Material

## Supplementary Figures


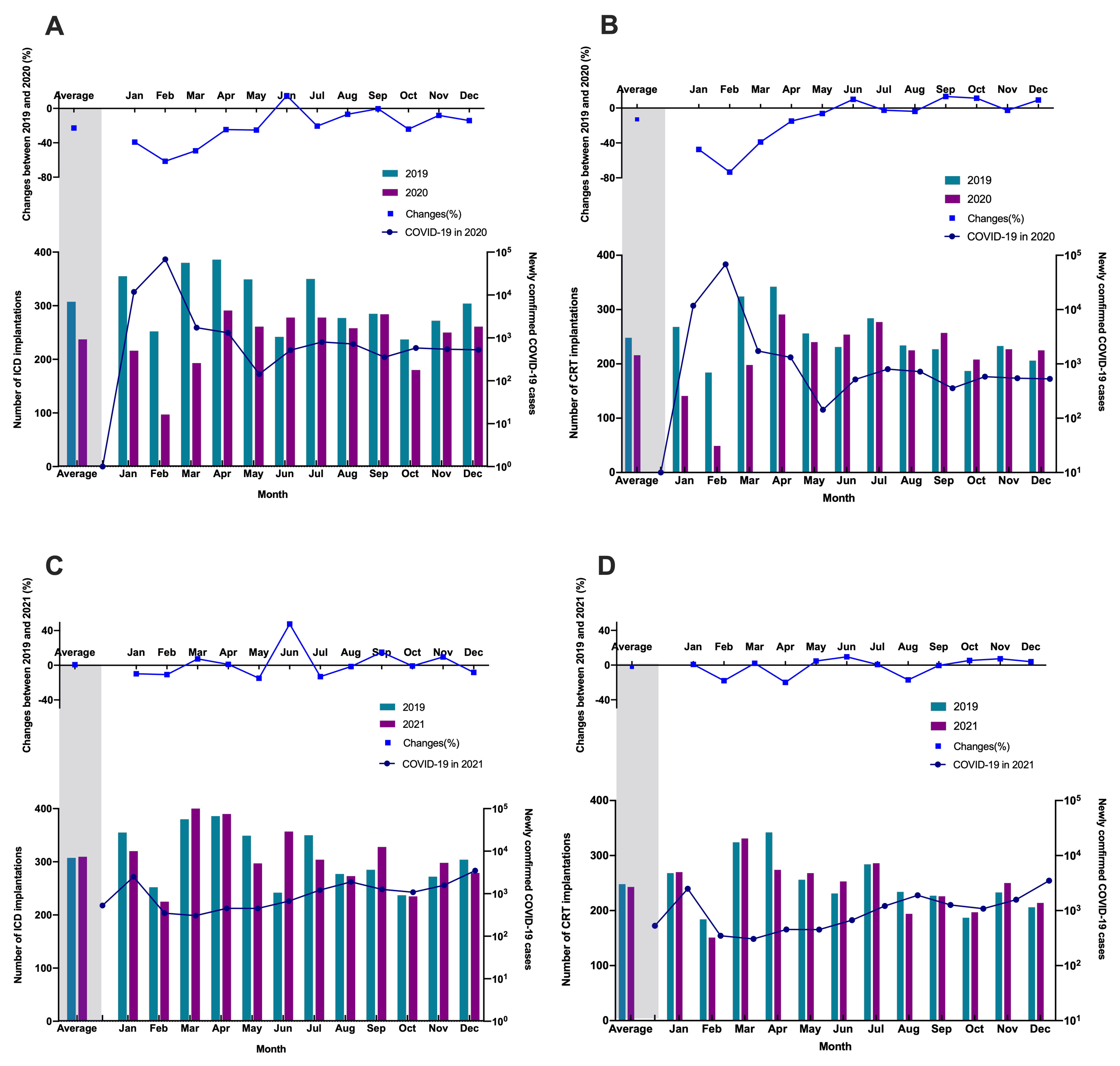


**Supplementary Figure 1.** The monthly number of ICD and CRT implantations in different periods and corresponding COVID-19 cases. A: ICD (2019 vs 2020, p<0.001); B: CRT (2019 vs 2020, p<0.001); C: ICD (2019 vs 2021, p=0.771); D:CRT (2019 vs 2021, p=0.419). CRT, cardiac resynchronization therapy; COVID-19, Coronavirus disease 2019; ICD, implantable cardiac defibrillator.

**
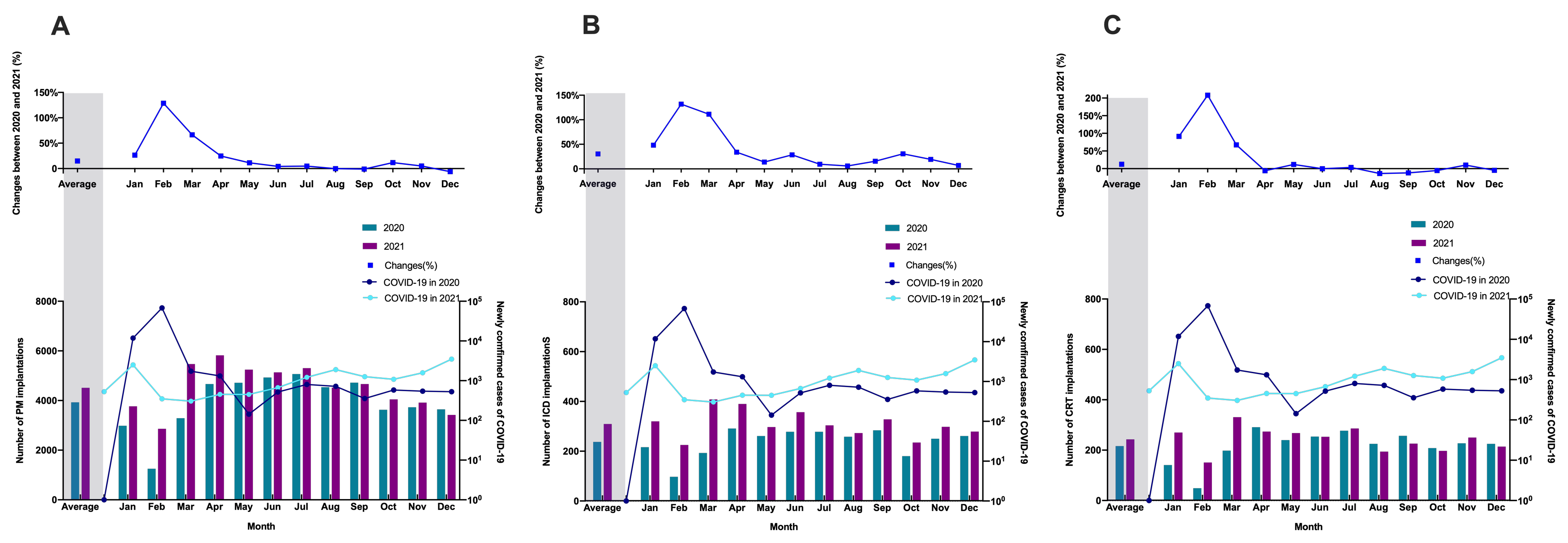
**

**Supplementary Figure 2**. The monthly number implantations and corresponding COVID-19 cases between 2020 and 2021. A: PM (2020 vs 2021, p<0.001); B: ICD (2020 vs 2021, p<0.001); C: CRT (2020 vs 2021, p<0.001). COVID-19, Coronavirus disease 2019; CRT, cardiac resynchronization therapy; ICD, implantable cardiac defibrillator. PM: pacemaker.

**Supplementary Table**

**Supplementary Table 1. Confirmed COVID-19 cases in 31 provinces in 2020 and 2021.**

| **Province** |  | **Confirmed COVID-19 cases** | |
| --- | --- | --- | --- |
|  |  | **In 2020** | **In 2021** |
| Anhui |  | 2940 | 993 |
| Beijing |  | 4897 | 987 |
| Fujian |  | 2011 | 513 |
| Gansu |  | 1917 | 182 |
| Guangdong |  | 4145 | 2046 |
| Guangxi |  | 900 | 264 |
| Guizhou |  | 1166 | 147 |
| Hainan |  | 117 | 171 |
| Hebei |  | 1346 | 373 |
| Henan |  | 1843 | 1299 |
| Heilongjiang |  | 1287 | 964 |
| Hubei |  | 4133 | 68149 |
| Hunan |  | 1818 | 1021 |
| Jilin |  | 1074 | 157 |
| Jiangsu |  | 2577 | 684 |
| Jiangxi |  | 1759 | 935 |
| Liaoning |  | 1648 | 351 |
| Neimenggu |  | 636 | 364 |
| Ningxia |  | 195 | 75 |
| Qinhai |  | 317 | 18 |
| Shandong |  | 2777 | 862 |
| Shanxi |  | 1219 | 224 |
| Shaanxi |  | 1084 | 507 |
| Shanghai |  | 3415 | 1516 |
| Xichuan |  | 6139 | 853 |
| Tianjing |  | 1637 | 309 |
| Xinjiang |  | 1980 | 980 |
| Yunnan |  | 2499 | 230 |
| Zhejiang |  | 5434 | 1306 |
| Chongqin |  | 936 | 590 |
| Xizang |  | 31 | 1 |
| Total |  | 87071 | 15243 |
